# Supplementary material for: Deletion of wfs1 Impairs Oligodendrocyte Precursor Cells Dorsal Distribution and Myelination Through the wfs1-hmgcs1 Axis in Zebrafish
Source: Biology (Basel). 2026 Aug 16;15(16):1402. doi: 10.3390/biology15161402 (PMC13510151; doi:10.3390/biology15161402)
Supplement: Supplementary file 1 [file biology-15-01402-s001.zip › File S1-Transcriptome sequencing data and differentially expressed genes (DEGs) in wfs1-- zebrafish at 5 dpf/2.KEGG/wfs1KOvsWT/ALL/src/dre04520.html]

dre04520


- dre:566841

- Up regulated genes

566841(1.91411798034227)

- dre:569566

- Down regulated genes

569566(-1.00953785382545)

- dre:571431

- Down regulated genes

571431(-6.42862549702529)

- dre:245702

- Up regulated genes

245702(1.27755220735994)

- Down regulated genes

245702\_1(-1.45433245048201)

- dre:335573

- Up regulated genes

335573\_1(4.21719504072814)

- dre:436718

- Up regulated genes

436718(1.34229427553561)

- Down regulated genes

436718\_1(-6.85571323128554)

- dre:436718

- Up regulated genes

436718(1.34229427553561)

- Down regulated genes

436718\_1(-6.85571323128554)

- dre:792699

- Down regulated genes

792699(-1.84389282152821)

- dre:792699

- Down regulated genes

792699(-1.84389282152821)

- dre:793158

- Up regulated genes

793158\_1(1.86160461223548)

- Down regulated genes

793158(-6.94943122774273)
- dre:556226

- Up regulated genes

556226\_1(6.36622858283894)

- dre:57934

- Up regulated genes

57934\_1(9.91304974129429)

- Down regulated genes

57934(-1.25539807424297)

- dre:57934

- Up regulated genes

57934\_1(9.91304974129429)

- Down regulated genes

57934(-1.25539807424297)

- dre:793158

- Up regulated genes

793158\_1(1.86160461223548)

- Down regulated genes

793158(-6.94943122774273)
- dre:556226

- Up regulated genes

556226\_1(6.36622858283894)
- dre:792699

- Down regulated genes

792699(-1.84389282152821)

- dre:793158

- Up regulated genes

793158\_1(1.86160461223548)

- Down regulated genes

793158(-6.94943122774273)
- dre:556226

- Up regulated genes

556226\_1(6.36622858283894)
- dre:792699

- Down regulated genes

792699(-1.84389282152821)

- dre:393527

- Up regulated genes

393527(1.22323032078635)

- dre:436718

- Up regulated genes

436718(1.34229427553561)

- Down regulated genes

436718\_1(-6.85571323128554)

- dre:321070

- Up regulated genes

101882911(3.50047007681359)

- dre:30429

- Up regulated genes

30429(3.98975094921512)

- Down regulated genes

30429\_2(-1.78714384880949)
- dre:677750

- Up regulated genes

677750(1.37913343503877)

- dre:378998

- Up regulated genes

564805\_1(4.21774027861677)

Close
